# AdamMC: A Model Checker for Petri Nets with Transits against Flow-LTL

**DOI:** 10.1007/978-3-030-53291-8_5

**Published:** 2020-06-16

**Authors:** Bernd Finkbeiner, Manuel Gieseking, Jesko Hecking-Harbusch, Ernst-Rüdiger Olderog

**Affiliations:** 8 Microsoft Research Lab, Redmond, WA USA; 9 University of Southern California, Los Angeles, CA USA; 10 Saarland University, Saarbrücken, Germany; 11 University of Oldenburg, Oldenburg, Germany

## Abstract

The correctness of networks is often described in terms of the individual data flow of components instead of their global behavior. In software-defined networks, it is far more convenient to specify the correct behavior of packets than the global behavior of the entire network. Petri nets with transits extend Petri nets and Flow-LTL extends LTL such that the data flows of tokens can be tracked. We present the tool AdamMC as the first model checker for Petri nets with transits against Flow-LTL. We describe how AdamMC can automatically encode concurrent updates of software-defined networks as Petri nets with transits and how common network specifications can be expressed in Flow-LTL. Underlying AdamMC is a reduction to a circuit model checking problem. We introduce a new reduction method that results in tremendous performance improvements compared to a previous prototype. Thereby, AdamMC can handle software-defined networks with up to 82 switches.

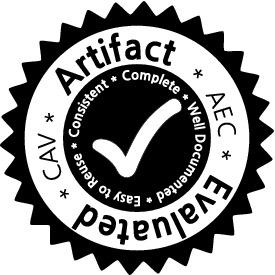



## Introduction

In networks, it is difficult to specify correctness in terms of the global behavior of the entire system. Instead, the individual *flow* of components is far more convenient to specify correct behavior. For example, loop and drop freedom can be easily specified for the flow of each packet. Petri nets and LTL lack this local view. Petri nets with transits and Flow-LTL have been introduced to overcome this restriction [10]. A transit relation is introduced to follow the *flow* induced by tokens. *Flow-LTL* is a temporal logic to specify both the *local* flow of data and the *global* behavior of markings. The global behavior as in Petri nets and LTL is still important for maximality and fairness assumptions. In this paper, we present the tool AdamMC^1^ as the first model checker for Petri nets with transits against Flow-LTL and its application to software-defined networking.

**Fig. 1.**
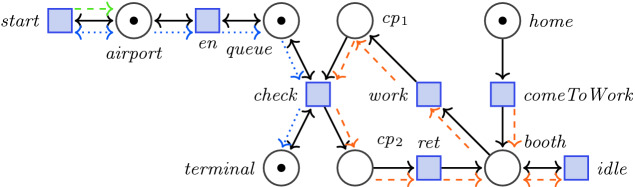
Access control at an airport modeled as Petri net with transits. Colored arrows display the transit relation and define flow chains to model the passengers.

In Fig. 1, we present an example of a Petri net with transits that models the security check at an airport where passengers are checked by a security guard. The number of passengers entering the airport is unknown in advance. Rather than introducing the complexity of an infinite number of tokens, we use a fixed number of tokens to model possibly infinitely many *flow chains*. This is done by the transit relation which is depicted with colored arrows.

The left-hand side of Fig. 1 models passengers who want to reach the terminal. There are three tokens in the places *airport*, *queue*, and *terminal*. Thus, transitions *start* and *en* are always enabled. Each firing of *start* creates a new flow chain as depicted by the green arrow. This models a new person arriving at the *airport*. Meanwhile, the double-headed blue arrow maintains all flow chains that are still in place *airport*. Passengers have to *en*ter the *queue* and wait until the security *check* is performed. Therefore, transition *en* continues every flow chain in *airport* to *queue*. Checking the passengers is carried out by transition *check* which becomes enabled if the security guard *work*s. Thus, passengers residing in *queue* have to wait until the guard *check*s them. Afterwards, they reach the *terminal*. The security guard is modeled on the right-hand side of Fig. 1. By firing *comeToWork* and thus moving the token in place *home*, her flow chain starts and she can repeatedly either *idle* or *work*, *check* passengers, and *ret*urn. Her transit relation is depicted in orange and models exactly one flow chain.

In Fig. 1, we define the checkpoints *cp*$_1$ and *cp*$_2$ and the *booth* as a security zone and require that passengers never enter the security zone and eventually reach the *terminal*. The flow formula  specifies this. AdamMC verifies the example from Fig. 1 against the formula  specifying that if passengers are checked regularly then they cannot access the security zone and eventually reach the terminal.

In this paper, we present AdamMC as a full-fledged tool. First, AdamMC can handle Petri nets with transits and Flow-LTL formulas in general. Second, AdamMC has an input interface for a concurrent update and a software-defined network and encodes both of them as a Petri nets with transits. Common assumptions on fairness and requirements for network correctness are also provided as Flow-LTL formulas. This allows users of the tool to model check the correctness of concurrent updates and to prevent packet loss, routing loops, and network congestion. Third, AdamMC provides algorithms to check safe Petri nets against LTL with *both* places and transitions as atomic propositions which makes it especially easy to specify fairness and maximality assumptions.

The tool reduces the model checking problem for safe Petri nets with transits against Flow-LTL to the model checking problem for safe Petri nets against LTL. We develop the new *parallel approach* to check global and local behavior in parallel instead of sequentially. This approach yields a tremendous speed-up for a few local requirements and realistic fairness assumptions in comparison to the sequential approach of a previous prototype [10]. In general, the parallel approach has worst-case complexity inferior to the sequential approach even though the complexities of both approaches are the same when using only one flow formula.

As last step, AdamMC reduces the model checking problem of safe Petri nets against LTL to a circuit model checking problem. This is solved by ABC [2, 4] with effective verification techniques like IC3 and bounded model checking. AdamMC verifies concurrent updates of software-defined networks with up to 38 switches (31 more than the prototype) and falsifies concurrent updates of software-defined networks with up to 82 switches (44 more than the prototype).

The paper is structured as follows: In Sect. 2, we recall Petri nets with transits and Flow-LTL. In Sect. 3, we outline the three application areas of AdamMC: checking safe Petri nets with transits against Flow-LTL, checking concurrent updates of software-defined networks against common assumptions and specifications, and checking safe Petri nets against LTL. In Sect. 4, we algorithmically encode concurrent updates of software-defined networks in Petri nets with transits. In Sect. 5, we introduce the parallel approach for the underlying circuit model checking problem. In Sect. 6, we present our experimental evaluation.

Further details can be found in the full paper [13].

## Petri Nets with Transits and Flow-LTL

A safe *Petri net with transits* $\mathscr {N}=(\mathscr {P},\mathscr {T},\mathscr {F}, In ,\varUpsilon )$ [10] contains the set of *places* $\mathscr {P}$, the set of *transitions* $\mathscr {T}$, the *flow relation* $\mathscr {F}\subseteq (\mathscr {P}\times \mathscr {T}) \cup (\mathscr {T}\times \mathscr {P})$, and the *initial marking* $In \subseteq \mathscr {P}$ as in safe Petri nets [27]. In a *safe* Petri net, reachable markings contain at most one token per place. The *transit relation* $\varUpsilon$ is for every transition $t \in \mathscr {T}$ of type $\varUpsilon (t) \subseteq ( pre ^{\mathscr {N}}(t)\cup \{\rhd \}) \times post ^{\mathscr {N}}(t)$. With $p\ \varUpsilon (t)\ q$, we define that firing transition *t* *transits* the flow in place *p* to place *q*. The symbol $\rhd$ denotes a *start* and $\rhd \ \varUpsilon (t)\ q$ defines that firing transition *t* *starts* a new flow for the token in place *q*. Note that the transit relation can split, merge, and end flows. A sequence of flows leads to a *flow chain* which is a sequence of the current place and the fired outgoing transition. Thus, Petri nets with transits can describe both the global progress of tokens and the local flow of data.

Flow-LTL [10] extends Linear-time Temporal Logic (LTL) and uses places and transitions as atomic propositions. It introduces $\mathbb {A}$ as a new operator which uses LTL to specify the flow of data for *all* flow chains. For Fig. 1, the formula  specifies that the guard performs at least one check. We call formulas starting with $\mathbb {A}$ *flow formulas*. Formulas around flow formulas specify the global progress of tokens in the form of markings and fired transitions to formalize maximality and fairness assumptions. These formulas are called *run formulas*. Often, Flow-LTL formulas have the form $run~formula \rightarrow flow~formula$.

**Fig. 2.**
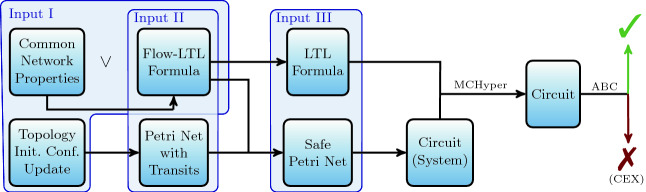
Overview of the workflow of $\textsc {AdamMC}$: The application areas of the tool are given by three different input domains: software-defined network/Flow-LTL (Input I), Petri nets with transits/Flow-LTL (Input II), and Petri nets/LTL (Input III). $\textsc {AdamMC}$ performs all unlabeled steps. MCHyper creates the final circuit which ABC checks to answer the initial model checking problem.

## Application Areas

AdamMC consists of modules for three application areas: checking safe Petri nets with transits against Flow-LTL, checking concurrent updates of software-defined networks against common assumptions and specifications, and checking safe Petri nets against LTL. The general architecture and workflow of the model checking procedure is given in Fig. 2. AdamMC is based on the tool Adam [14].

**Petri Nets with Transits.  ** Petri nets with transits follow the progress of tokens and the flow of data. Flow-LTL allows to specify requirements on both. For Petri nets with transits and Flow-LTL (Input II), AdamMC extends a parser for Petri nets provided by APT [30], provides a parser for Flow-LTL, and implements two reduction methods to create a safe Petri net and an LTL formula. The sequential approach is outlined in [10] and the parallel approach in Sect. 5.

**Software-Defined Networks.  ** Concurrent updates of software-defined networks are the second application area of AdamMC. The tool automatically encodes an initially configured network topology and a concurrent update as a Petri net with transits. The concurrent update renews the forwarding table. We provide parsers for the *network topology*, the *initial configuration*, the *concurrent update*, and Flow-LTL (Input I). In Sect. 4, we present the creation of a Petri net with transits from the input and Flow-LTL formulas for *common network properties* like *connectivity*, *loop freedom*, *drop freedom*, and *packet coherence*.

**Petri Nets.** AdamMC supports the model checking of safe Petri nets against LTL with both places *and* transitions as atomic propositions. It provides dedicated algorithms to check *interleaving-maximal* runs of the system. A run is interleaving-maximal if a transition is fired whenever a transition is enabled. Furthermore, AdamMC allows a concurrent view on runs and can check *concurrency-maximal* runs which demand that each subprocess of the system has to progress maximally rather than only the entire system. State-of-the-art tools like LoLA [32] and ITS-Tools [29] are restricted to interleaving-maximal runs and places as atomic propositions. For Petri net model checking (Input III), we allow Petri nets in APT and PNML format as input and provide a parser for LTL formulas.

The construction of the circuit in Aiger format [3] is defined in [11]. MCHyper [15] is used to create a circuit from a given circuit and an LTL formula. This circuit is given to ABC [2, 4] which provides a toolbox of modern hardware verification algorithms like IC3 and bounded model checking to decide the initial model checking question. As output for all three modules, AdamMC transforms a possible counterexample (CEX) from ABC into a counterexample to the Petri net (with transits) and visualizes the net with Graphviz and the dot language [9]. When no counterexample exists, AdamMC verified the input successfully.

## Verifying Updates of Software Defined Networks

We show how AdamMC can check concurrent updates of realistic examples from software-defined networking (SDN) against typical specifications [19]. SDN [6, 25] separates the *data plane* for forwarding packets and the *control plane* for the routing configuration. A central controller initiates updates which can cause problems like routing loops or packet loss. AdamMC provides an input interface to automatically encode software-defined networks and concurrent updates of their configuration as Petri nets with transits. The tool checks requirements like loop and drop freedom to find erroneous updates before they are deployed.

### Network Topology, Configurations, and Updates

A *network topology* *T* is an undirected graph $T=( Sw , Con )$ with *switches* as vertices and *connections* between switches as edges. Packets enter the network at *ingress* switches and they leave at *egress* switches. *Forwarding* rules are of the form $\texttt {x.fwd(y)}$ with $\texttt {x}, \texttt {y} \in Sw$. A concurrent *update* has the following syntax:

$$ \begin{array}{lll} \text {switch update}\!\!\!\! &{}{::=}\,\texttt {upd(x.fwd(y/z))\,} | \texttt {\,upd(x.fwd(y/-))\,} | \texttt {\,upd(x.fwd(-/z))}\\ \text {sequential update}\!\!\!\! &{}{::=}\,\texttt {(}\text {update } \texttt {>>} \text { update } \texttt {>>} \text { ... } \texttt {>>} \text { update}{} \texttt {)} \\ \text {parallel update}\!\!\!\! &{}{::=}\,\texttt {(} \text {update } \texttt {||} \text { update } \texttt {||} \text { ... } \texttt {||} \text { update} \texttt {)} \\ \text {update}\!\!\!\! &{}{::=}\,\text {switch update}\, | \,\text {sequential update}\, | \,\text {parallel update}\\ \end{array} $$

where a switch update can renew the forwarding rule of switch x from switch z to switch y, introduce a new forwarding rule from switch x to switch y, or remove an existing forwarding rule from switch x to switch z.

### Data Plane and Control Plane as Petri Net with Transits

For a network topology $T = ( Sw , Con )$, a set of *ingress* switches, a set of *egress* switches, an initial *forwarding* table, and a concurrent $update$, we show how data and control plane are encoded as Petri net with transits. Switches are modeled by tokens remaining in corresponding places s whereas the flow of packets is modeled by the transit relation $\varUpsilon$. Specific transitions $i_\texttt {s}$ model ingress switches where new data flows begin. Tokens in places of the form x.fwd(y) configure the forwarding. Data flows are extended by firing transitions $\texttt {(x,y)}$ corresponding to configured forwarding without moving any tokens. Thus, we model any order of newly generated packets and their forwarding. Assuming that each existing direction of a connection between two switches is explicitly given in $Con$, we obtain Algorithm 1 which calls Algorithm 2 to obtain the control plane.

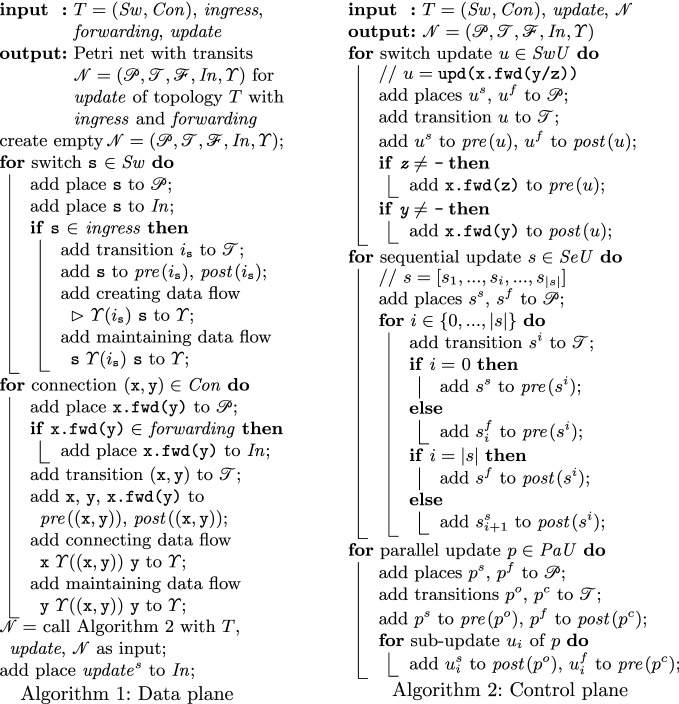



For the $update$, let $SwU$ be the set of switch updates in it, $SeU$ the set of sequential updates in it, and $PaU$ the set of parallel updates in it. Depending on $update$’s type, it is also added to the respective set. The subnet for the *update* has an empty transit relation but moves tokens from and to places of the form $\texttt {x.fwd(y)}$. Tokens in these places correspond to the forwarding table. The order of the switch updates is defined by the nesting of sequential and parallel updates. The *update* is realized by a specific token moving through unique places of the form $u^s, u^f, s^s, s^f, p^s, p^f$ for start and finish of each switch update $u\in SwU$, each sequential update $s\in SeU$, and each parallel update $p\in PaU$. A parallel update temporarily increases the number of tokens and reduces it upon completion to one. Algorithm 2 defines the update behavior between start and finish places and connects finish and start places depending on the subexpression structure.

**Fig. 3.**
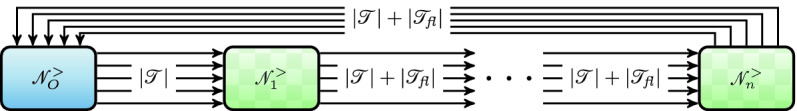
Overview of the *sequential approach*: Each firing of a transition of the original net is split into first firing a transition in the subnet for the run formula and subsequently firing a transition in each subnet tracking a flow formula. The constructed LTL formula skips the additional steps with until operators.

**Fig. 4.**
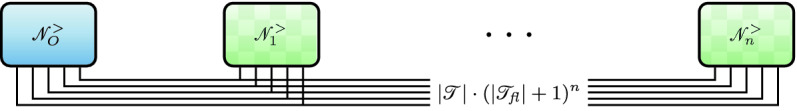
Overview of the *parallel approach*: The $n$ subnets are connected such that for every transition $t\in \mathscr {T}$ there are $(|\varUpsilon (t)|+1)^n$ transitions, i.e., there is one transition for every combination of which transit of $t$ (or none) is tracked by which subnet. We use until operators in the constructed LTL formula to only skip steps not involving the tracking of the guessed chain in the flow formula.

### Assumptions and Requirements

We use the run formula  to assume weak fairness for every transition *t* in our encoding $\mathscr {N}$. Transitions, which are always enabled after some point, are ensured to fire infinitely often. Thus, packets are eventually forwarded and the routing table is eventually updated. We use flow formulas to test specific requirements for all packets. Connectivity () ensures that all packets reach an egress switch. Packet coherence () tests that packets are either routed according to the initial or final configuration. Drop freedom () forbids dropped packets whereas loop freedom () forbids routing loops. We combine run and flow formula into *fairness* $\rightarrow$ *requirement*.

## Algorithms and Optimizations

Central to model checking a Petri net with transits $\mathscr {N}$ against a Flow-LTL formula $\varphi$ is the reduction to a safe Petri net $\mathscr {N}^{>}$ and an LTL formula $\varphi ^{>}$. The infinite state space of the Petri net with transits due to possibly infinitely many flow chains is reduced to a finite state model. The key idea is to guess and track a violating flow chain for each flow subformula $\mathbb {A}\,\psi _i$, for $i\in \{1,\ldots ,n\}$, and to only once check the equivalent future of flow chains merging into a common place.

AdamMC provides two approaches for this reduction: Fig. 3 and Fig. 4 give an overview of the *sequential* approach and the *parallel* approach, respectively. Both algorithms create one subnet $\mathscr {N}^{>}_{i}$ for each flow subformula $\mathbb {A}\,\psi _i$ to track the corresponding flow chain and have one subnet $\mathscr {N}^{>}_O$ to check the run part of the formula. The places of $\mathscr {N}^{>}_O$ are copies of the places in $\mathscr {N}$ such that the current state of the system can be memorized. The subnets $\mathscr {N}^{>}_{i}$ also consist of the original places of $\mathscr {N}$ but only use one token (initially residing on an additional place) to track the current state of the considered flow chain. The approaches differ in how these nets are connected to obtain $\mathscr {N}^{>}$.

**Sequential Approach.  ** The places in each subnet $\mathscr {N}^{>}_{i}$ are connected with one transition for each transit ($\mathscr {T}_{ fl }=\bigcup _{t\in \mathscr {T}} \varUpsilon (t)$). An additional token iterates sequentially through the subnets to activate or deactivate the subnet. This allows each subnet to track a flow chain corresponding to firing a transition in $\mathscr {N}^{>}_O$. The formula $\varphi ^{>}$ takes care of these additional steps by means of the until operator: In the run part of the formula, all steps corresponding to moves in a subnet $\mathscr {N}^{>}_{i}$ are skipped and, for each subformula $\mathbb {A}\,\psi _{i}$, all steps are skipped until the next transition of the corresponding subnet is fired which transits the tracked flow chain. This technique results in a polynomial increase of the size of the Petri net and the formula: $\mathscr {N}^{>}$ has $\mathscr {O}(|\mathscr {N}|\cdot n + |\mathscr {N}|)$ places and $\mathscr {O}(|\mathscr {N}|^3\cdot n + |\mathscr {N}|)$ transitions and the size of $\varphi ^{>}$ is in $\mathscr {O}(|\mathscr {N}|^3\cdot n \cdot |\varphi | + |\varphi |)$. We refer to [11] for formal details.

**Parallel Approach.  ** The $n$ subnets are connected such that the current chain of each subnet is tracked simultaneously while firing an original transition $t\in \mathscr {T}$. Thus, there are $(|\varUpsilon (t)|+1)^n$ transitions. Each of these transitions stands for exactly one combination of which subnet is tracking which (or no) transit. Hence, firing one transition of the original net is directly tracked in one step for all subnets. This significantly reduces the complexity of the run part of the constructed formula, since no until operator is needed to skip sequential steps. A disjunction over all transitions corresponding to an original transition suffices to ensure correctness of the construction. Transitions and next operators in the flow parts of the formula still have to be replaced by means of the until operator to ensure that the next step of the tracked flow chain is checked at the corresponding step of the global timeline of $\varphi ^{>}$. In general, the parallel approach results in an exponential blow-up of the net and the formula: $\mathscr {N}^{>}$ has $\mathscr {O}(|\mathscr {N}|\cdot n + |\mathscr {N}|)$ places and $\mathscr {O}(|\mathscr {N}|^{3n}+|\mathscr {N}|)$ transitions and the size of $\varphi ^{>}$ is in $\mathscr {O}(|\mathscr {N}|^{3n}\cdot |\varphi | + |\varphi |)$. For the practical examples, however, the parallel approach allows for model checking Flow-LTL with few flow subformulas with a tremendous speed-up in comparison to the sequential approach. Formal details are in the full version of the paper [13].

**Optimizations.  ** Various optimizations parameters can be applied to the model checking routine described in Sect. 3 to tweak the performance. Table 1 gives an overview of the major parameters.

**Table 1.**
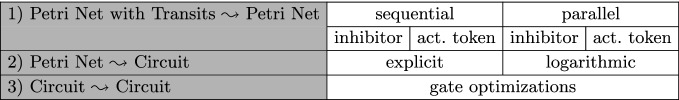
Overview of optimization parameters of AdamMC: The three reduction steps depicted in the first column can each be executed by different algorithms. The first step allows to combine the optimizations of the first and second row.

We found that the versions of the sequential and the parallel approach with inhibitor arcs to track flow chains are generally faster than the versions without. Furthermore, the reduction step from a Petri net into a circuit with logarithmically encoded transitions had oftentimes better performance than the same step with explicitly encoded transitions. However, several possibilities to reduce the number of gates of the created circuit worsened the performance of some benchmark families and improved the performance of others. Consequently, all parameters are selectable by the user and a script is provided to compare different settings. An overview of the selectable optimization parameters can be found in the documentation of AdamMC [12]. Our main improvement claims can be retraced by the case study in Sect. 6.

**Table 2.**
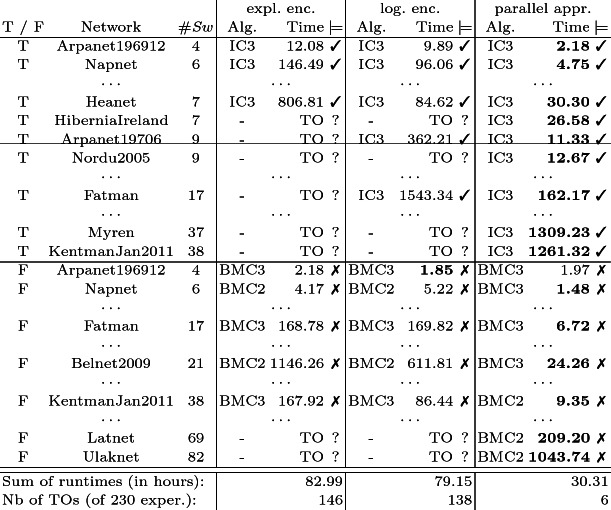
We compare the explicit and logarithmic encoding of the sequential approach with the parallel approach. The results are the average over five runs from an Intel i7-2700K CPU with 3.50 GHz, 32 GB RAM, and a timeout (TO) of 30  min. The runtimes are given in seconds.

## Evaluation

We conduct a case study based on SDN with a corresponding artifact [16]. The performance improvements of AdamMC compared to the prototype [10] are summarized in Table 2. For realistic software-defined networks [19], one ingress and one egress switch are chosen at random. Two forwarding tables between the two switches and an update from the first to the second configuration are chosen at random. AdamMC verifies that the update maintained *connectivity* between ingress and egress switch. The results are depicted in rows starting with T. For rows starting with F, we required *connectivity* of a random switch which is not in the forwarding tables. AdamMC falsified this requirement for the update.

The prototype implementation based on an *explicit encoding* can verify updates of networks with 7 switches and falsify updates of networks with 38 switches. We optimize the explicit encoding to a *logarithmic encoding* and the number of switches for which updates can be verified increases to 17. More significantly, the *parallel approach* in combination with the logarithmic encoding leads to tremendous performance gains. The performance gains of an approach with inferior worst-case complexity are mainly due to the smaller complexity of the LTL formula created by the reduction. The encoding of SDN requires fairness assumptions for each transition. These assumptions (encoded in the run part of the formula) experience a blow-up with until operators by the sequential approach but only need a disjunction in the parallel approach. Hence, the size of networks for which AdamMC can verify updates increases to 38 switches and the size for which it can falsify updates increases to 82 switches. For rather small networks, the tool needs only a few seconds to verify and falsify updates which makes it a great option for operators when updating networks.

## Related Work

We refer to [21] for an introduction to SDN. Solutions for correctness of updates of software-defined networks include *consistent updates* [7, 28], *dynamic scheduling* [17], and *incremental updates* [18]. Both explicit and SMT-based model checking [1, 5, 22, 23, 26, 31] is used to verify software-defined networks. Closest to our approach are models of networks as Kripke structures to use model checking for synthesis of correct network updates [8, 24]. The model checking subroutine of the synthesizer assumes that each packet sees at most one updated switch. Our model checking routine does not make such an assumption.

There is a significant number of model checking tools (e.g., [29, 32]) for Petri nets and an annual model checking contest [20]. AdamMC is restricted to safe Petri nets whereas other tools can handle bounded and colored Petri nets. At the same time, only AdamMC accepts LTL formulas with places *and* transitions as atomic propositions. This is essential to express fairness in our SDN encoding.

## Conclusion

We presented the tool AdamMC with its three application domains: checking safe Petri nets with transits against Flow-LTL, checking concurrent updates of software-defined networks against common assumptions and specifications, and checking safe Petri nets against LTL. New algorithms allow AdamMC to model check software-defined networks of realistic size: it can verify updates of networks with up to 38 switches and can falsify updates of networks with up to 82 switches.

## Footnotes

1 AdamMC is available online at https://uol.de/en/csd/adammc [12].
